# Bilateral striatal necrosis associated with enterovirus infection

**DOI:** 10.1590/0037-8682-0044-2021

**Published:** 2021-04-12

**Authors:** Ferdinand Dueñas Cabrera, Bruno Niemeyer de Freitas Ribeiro, Edson Marchiori

**Affiliations:** 1 Universidade Federal do Rio de Janeiro, Departamento de Radiologia, Rio de Janeiro, RJ, Brasil.; 2 3D Diagnóstico por imagem, Hospital Casa de Portugal, Rio de Janeiro, RJ, Brasil.

A 20-year-old man was admitted to the hospital due to hypotonia, dystonic movements, and dysarthria. The patient’s symptoms began and had been progressing since the age of 5, when he experienced an episode of enteroviral encephalitis.

**FIGURE 1:**
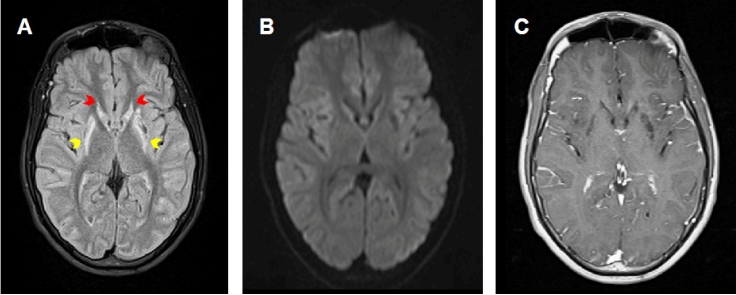
**(A)** Axial fluid attenuation inversion recovery sequence shows bilateral high signal intensity of the caudate nuclei (red arrows) and putamina (yellow arrows). **(B)** Diffusion-weighted imaging shows no restricted diffusion. **(C)** T1-weighted images obtained after contrast injection demonstrate no contrast enhancement.

The patient’s family history included no relevant information. His blood count, biochemistry, and cerebrospinal fluid at the time of admission were unremarkable. Laboratory investigation yielded negative findings for Huntington’s disease, neuroacanthocytosis, Wilson’s disease, and mitochondrial encephalopathies. Brain magnetic resonance imaging showed bilateral volume loss, and high signal intensity of the caudate nuclei and putamina on a fluid attenuation inversion recovery sequence, with no enhancement after contrast injection (Figure 1). Given his clinical history of viral encephalitis associated with progressive neurological symptoms and imaging findings, enterovirus-associated bilateral striatal necrosis (BSN) was the most likely diagnosis.

BSN is a rare neurological condition affecting the neostriata (putamina and caudate nuclei) and is defined in histopathology by initial tissue swelling, followed by degeneration and necrosis^1^. BSN has a wide variety of clinical manifestations, the most important being movement disorders^1^. It has multiple etiologies, including infectious, inflammatory, autoimmune, and metabolic conditions. The most common infectious agent related to BSN is *Mycoplasma pneumoniae*; less common agents include *Streptococci*, measles virus, human herpesvirus 6, rotavirus, and herpes simplex virus 1^1^. Enteroviruses are neurotropic and neurovirulent, and thus can cause a range of neurological manifestations, including encephalitis, meningitis, and BSN^2^. The diagnosis of BSN is challenging; adequate correlation of the clinical presentation, imaging, and laboratory findings is essential to establish it.
